# Lateralized occipital degeneration in posterior cortical atrophy predicts visual field deficits

**DOI:** 10.1016/j.nicl.2017.01.012

**Published:** 2017-01-17

**Authors:** Rebecca S Millington, Merle James-Galton, Mari N Maia Da Silva, Gordon T Plant, Holly Bridge

**Affiliations:** a Oxford Centre for fMRI of the Brain (FMRIB), Nuffield Department of Clinical Neurosciences, University of Oxford, Oxford, UK; b National Hospital for Neurology and Neurosurgery, London, UK; c Moorfields Eye Hospital, London, UK

**Keywords:** Posterior cortical atrophy, Hemianopia, Visual dysfunction, Occipital lobe, Magnetic resonance imaging

## Abstract

**Background:**

Posterior cortical atrophy (PCA), the visual variant of Alzheimer's disease, leads to high-level visual deficits such as alexia or agnosia. Visual field deficits have also been identified, but often inconsistently reported. Little is known about the pattern of visual field deficits or the underlying cortical changes leading to this visual loss.

**Methods:**

Multi-modal magnetic resonance imaging was used to investigate differences in gray matter volume, cortical thickness, white matter microstructure and functional activity in patients with PCA compared to age-matched controls. Additional analyses investigated hemispheric asymmetries in these metrics according to the visual field most affected by the disease.

**Results:**

Analysis of structural data indicated considerable loss of gray matter in the occipital and parietal cortices, lateralized to the hemisphere contralateral to the visual loss. This lateralized pattern of gray matter loss was also evident in the hippocampus and parahippocampal gyrus. Diffusion-weighted imaging showed considerable effects of PCA on white matter microstructure in the occipital cortex, and in the corpus callosum. The change in white matter was only lateralized in the occipital lobe, however, with greatest change in the optic radiation contralateral to the visual field deficit. Indeed, there was a significant correlation between the laterality of the optic radiation microstructure and visual field loss.

**Conclusions:**

Detailed brain imaging shows that the asymmetric visual field deficits in patients with PCA reflect the pattern of degeneration of both white and gray matter in the occipital lobe. Understanding the nature of both visual field deficits and the neurodegenerative brain changes in PCA may improve diagnosis and understanding of this disease.

## Highlights

• Patients with posterior cortical atrophy show asymmetric visual field deficits manifesting as hemianopia.
• Both gray and white matter show lateralized degeneration corresponding to the most affected visual field.
• Laterality of microstructure in the optic radiation correlates with visual field loss.

## 1 Introduction

Posterior cortical atrophy (PCA) is a progressive neurodegenerative disease, often described as the visual variant of Alzheimer disease (AD). The disorder is characterized by an early presentation of higher order visual processing deficits and mild memory impairments, with memory dysfunction occurring with progression of the disease (Crutch et al., 2012).

The most commonly reported impairments in PCA are higher-level visual problems such as alexia or visuospatial processing, however hemianopia has been reported in a number of studies. Reported incidence of hemianopia in PCA is between 10 and 20% in most studies (Schmidtke et al., 2005, McMonagle et al., 2006, Whitwell et al., 2007, Andrade et al., 2010), the highest being 80% (Kaeser et al., 2015). Tang-Wai et al. (2004) found hemianopia to be one of the most prevalent visual deficits in their cohort of PCA patients, with 35% exhibiting a homonymous field deficit, and a further 12.5% having less consistent field deficits. As such, they proposed that visual field deficits should be considered a core clinical feature in diagnosing PCA. However, in spite of the number of studies reporting hemianopia in PCA patients, the prevalence, and indeed the existence of visual field deficits in these patients remains controversial. Crutch et al. (2012) state that hemianopia is often misdiagnosed in PCA patients due to the presence of higher-level visual deficits such as hemispatial neglect, and older studies of the visual deficits in PCA patients have failed to report any visual field deficits (Benson et al., 1988, Victoroff et al., 1994). Mendez et al. (2002) actively screened out patients with visual field deficits when constructing a cohort in which to study the clinical characteristics of PCA, citing intact primary visual function as a core diagnostic feature. Indeed many studies investigating the visual deficits characteristic of PCA do not include visual field testing in their study battery, although field deficits may be incidentally reported. Where visual field loss has been accounted for, visual confrontation is often used (Benson et al., 1988, Andrade et al., 2010), which is not as sensitive as perimetry in detecting field deficits (Johnson and Baloh, 1991).

While recent studies have attempted to systematically quantify visual fields in PCA patients (Formaglio et al., 2009, Pelak et al., 2011), little is known of the pattern of degeneration underlying this visual deficit, and its relationship to homonymous hemianopia due to V1 damage. Neuroimaging in PCA has indicated bilateral loss of gray matter in the occipital, parietal, and posterior temporal lobes, and the right hemisphere appears to be more severely affected (Whitwell et al., 2007, Migliaccio et al., 2012a, Migliaccio et al., 2012b), particularly in patients with more ‘dorsal’ symptoms (Migliaccio et al., 2012c). Similarly, case studies indicate white matter microstructure is also affected in the parietal and occipital lobes (Duning et al., 2009) and may be present throughout the major white matter pathways involved in visuo-spatial processing (Migliaccio et al., 2012c).

The aim of the present study was firstly to shed light on the pathological basis of the visual field defects in this group of patients and secondly to use the existence of the defect as a marker of asymmetry in the involvement of the two hemispheres. Comparisons between symmetrical structures in the same individual can prove more sensitive in revealing change than between cases and controls. Thus, we apply multimodal neuroimaging to test the hypothesis that asymmetric degeneration of gray and white matter in the occipital cortex underlies the lateralized visual field deficits present in these PCA patients.

## 2 Methods

### 2.1 Subjects

Ten patients with a diagnosis of PCA were recruited from the National Hospital for Neurology and Neurosurgery. For a diagnosis of PCA, patients needed to demonstrate progressive impairment of posterior cortical function with relative preservation of memory and other cognitive functions. The cases all fulfilled the diagnostic criteria proposed by Tang-Wai et al. (2004) with the exception that the presence of a visual field defect was a necessary condition for inclusion. The patients were all recruited from a neuro-ophthalmology clinic and therefore were often referred due to presence of a visual field deficit. Furthermore, all patients who attend the clinic have automated fields performed as part of standard work up, so deficits are more likely to be detected. Patient ages ranged from 53 to 77 years (median 70), and included six females and four males. All patients displayed an asymmetric visual field (VF) deficit, with eight having the greatest VF loss in the left hemifield. The longest time since diagnosis of PCA was 6 years (P01), and the shortest was three months (P06), although in most cases PCA symptoms had been present for some time before diagnosis. Estimated time since onset of symptoms in each patient is given in Table 1. Prior to inclusion in the study, patients underwent neuropsychological assessment to ensure memory deficits did not exceed mild cognitive impairment. A summary of patient details is given in Table 1.

**Table 1:** Summary of patient details.

	Age^a^	Sex	Duration^b^	HVF mean deviation		
				Left	Right	Greater VF loss
P01	66	M	6	28.37	21.08	Left
P02	77	F	6	0.92	19.02	Right
P03	72	M	7	19.5	4.37	Left
P04	65	F	2	29.33	17.62	Left
P05	53	M	6	19.23	0.77	Left
P06	68	F	1	29.40	16.77	Left
P07	72	F	3	28.44	13.67	Left
P08	70	F	3	11.40	5.15	Left
P09	76	F	4	22.28	2.84	Left
P10	69	M	4	5.15	28.60	Right

a Age when scanned.

b Time from estimated onset of PCA symptoms to scan in years.

Data collected from ten healthy controls were used to compare with the PCA patients (age range 57–78, median age 73, 4 females). Four of these were scanned for a previous study, and six were scanned as part of Oxford Project to Investigate Memory and Ageing (OPTIMA). For the fMRI analyses, a slightly different set of control data was used as fMRI data was not collected in OPTIMA (*n* = 9, age range 31–74, median age 52, 4 females).

All participants gave written informed consent prior to participation, and the study was granted ethical approval from the *Oxfordshire National Health Service Research Ethics Committee* (*08/H0605/156*).

### 2.2 Perimetry

**Fig. 1:**
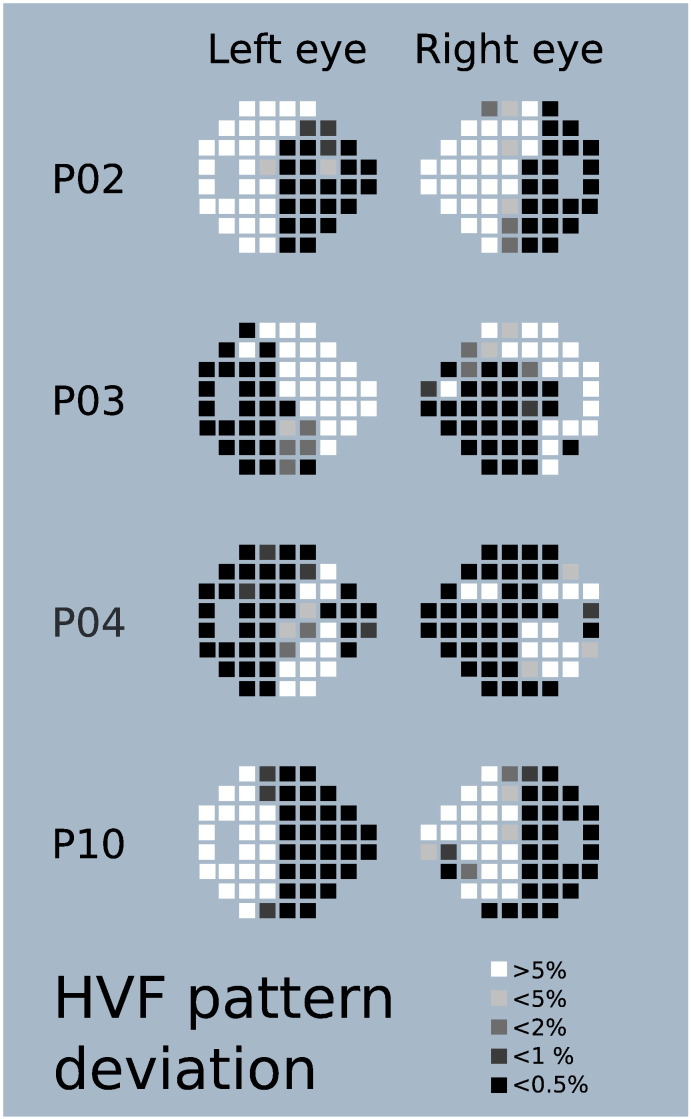
Examples of visual field deficits in patients with PCA. In P02 and P10 the deficit is limited to a hemifield, while in P03 and P04 both hemifields are affected to an extent. The shade of the box indicates the number of trials seen at each location after correction for generalised decreases in visual sensitivity with white being areas most sensitive to visual stimuli and black being locations with the greatest visual field loss.

Patients with a diagnosis of PCA with no evidence of any other ophthalmic or neurological disorder were screened for inclusion in the study. Humphrey visual fields (HVF) were acquired with a Zeiss Humphrey Field Analyzer, using central 24–2 threshold test and SITA-Standard strategy. Patients who demonstrated consistent homonymous visual field deficits were recruited for the study. Visual field examples are shown in Fig. 1. Half of the patients showed VF loss in both hemifields, although in all cases the deficit was greater in the left hemifield. The other five patients had either hemianopic or quadrantanopic VF loss, affecting the left in three patients and the right in two patients. The field loss was less dense than is generally seen in hemianopia acquired following stroke or trauma, and somewhat less congruent between eyes. HVF results may, however, be affected by the confounding higher level cognitive deficits experienced by PCA patients, which may interfere with their ability to perform the Humphrey field test. False positives were low for all patients (mean 2.20%; SD 2.26), and fixation losses were rare. False negatives were more variable (mean 22.95%; SD 20.56) ranging from 0% to 59% (P04 and P06) and 66% for P01. A high level of false negatives implies that the detection thresholds rise during the test. While most patients had lower numbers of false negatives, the extensive false negatives in these three patients mean that the field loss may be less extensive in these cases than the HVF results suggest, and that these patients exhibited a form of visual fatigue.

### 2.3 Neglect

All patients were tested for the presence of neglect. Two methods were employed: line bisection (Schenkenberg et al., 1980) and cancellation tasks (Gauthier et al., 1989).

### 2.4 Imaging

Functional MRI, diffusion-weighted and high-resolution T1 structural scans were acquired for all participants using a 3T Siemens Trio at the Oxford Centre for Clinical Magnetic Resonance Research. Two sets of whole brain diffusion weighted data were acquired. The diffusion weighting was isotropically distributed along 60 directions, b-value 1000 s/mm^2^, 65 slices; 2 mm^3^ voxels; TR = 9.3 s, TE = 94 ms. Volumes without diffusion weighting (b-value 0 s/mm^2^) were acquired for both sets of data. T1-weighted structural scans were acquired axially at a resolution of 1 mm^3^, 192 slices, TR = 2.04 s, TE = 4.7 ms.

For all MRI analysis, PCA patients were analysed according to which hemisphere was most atrophied, with the most affected hemisphere termed the *contralateral* hemisphere (being contralateral to the largest VF loss) and the other hemisphere termed the *ipsilateral* hemisphere. Data for P02 and P10 were therefore flipped in the x-axis so that the right hemisphere was the hemisphere contralateral to the greatest VF deficit in all patients. All MRI images were pre-processed and analysed using Oxford Centre for Functional MRI of the Brain's (FMRIB) software library (FSL; Smith et al., 2004).

#### 2.4.1 White matter

Diffusion data were pre-processed and analysed using FMRIB's diffusion toolbox (FDT). Diffusion images were corrected for distortions resulting from eddy-currents and head movements. Images were skull stripped using the brain extraction tool (Smith, 2002) and the two sets of data were averaged. Diffusion tensor models were applied using DTIFIT to obtain images of fractional anisotropy (FA) and mean diffusivity (MD) levels. Tract-based spatial statistics (TBSS, Smith et al., 2006) was used to allow voxelwise comparisons across subject groups. TBSS projects FA and MD data from all participants onto a mean FA tract skeleton, before applying voxelwise cross-subject statistics. PCA patients were compared to controls using randomise (Winkler et al., 2014) with Threshold-Free Cluster Enhancement (TFCE) applied. Hemispheric asymmetry in the PCA patients was computed first by generating a symmetric mean FA image, then projecting each subject's FA data onto that symmetric image. A flipped version of the subject's FA data was then also projected onto the symmetric image and the difference between the two FA images was taken as the asymmetry image. A1-sample group mean test was then used to determine the level of asymmetry across the group. Finally, the optic radiation was defined as an ROI using the Juelich histological atlas (Bürgel et al., 2006). The atlas ROI was thresholded at 20%, symmetrized and skeletonized. The mean FA and MD were then calculated for voxels in the skeletonized optic radiation ROI in each hemisphere for each subject.

#### 2.4.2 Gray matter

Gray matter damage was assessed using FSL-VBM software (Douaud et al., 2007, Good et al., 2001). Brain extracted structural images were tissue-type segmented, non-linearly registered to MNI 152 standard space (Andersson et al., 2007), concatenated, and averaged. They were then flipped along the x-axis and then the two mirror image averages were re-averaged to create a symmetric study specific template. Gray matter segmentations for each subject were then non-linearly registered to the study specific template. Resulting registered gray matter images were then smoothed with an isotropic Gaussian kernel with a sigma of 3 mm. A voxelwise general linear model was applied using permutation-based non-parametric testing to investigate differences in gray matter volume between PCA patients and controls. TFCE was used to determine regions with significant differences. Hemispheric asymmetry was calculated in the same way as the white matter in that original and flipped versions of the subject's gray matter segmentation were projected onto the study template. The difference between these two gray matter images was taken as the asymmetry image. *A*1-sample group mean test was then used to determine the level of asymmetry across the group.

Freesurfer (http://surfer.nmr.mgh.harvard.edu/) was used to analyze cortical thickness. This method extracts the pial and white matter surfaces on T1-weighted structural scans and computes the distance between them in order to measure the thickness of the cortical ribbon in between (Fischl and Dale, 2000). Initial outputs from the standard Freesurfer cortical reconstruction processing stream were inspected and the ventricles manually segmented to correct for reconstruction errors due to enlarged ventricles, before completing reconstruction. To investigate the visual areas most affected by gray matter loss, three particular regions of interest were chosen for further analysis in the visual cortex: V1, V2, and hMT+. Labels for each of these areas were used from the automatic brain segmentation in Freesurfer, using the Brodmann atlas. These cytoarchitectonically defined regions are identified on the basis of cortical folding patterns (Fischl et al., 2008). Average cortical thickness measures were extracted for each of these areas for each subject.

#### 2.4.3 Functional imaging

Functional MRI was acquired while visual stimuli were presented on a screen behind the participant's head, viewed through a mirror. This ‘motion’ scan, designed to activate motion sensitive regions of the visual cortex contrasted moving dots with stationary dots in a block design. Since many patients with hemianopia due to V1 damage retain neural activity to visual motion stimulation in extrastriate regions (Ajina et al., 2015), a similar stimulus was employed here to determine residual visual function. An 8° diameter patch of black dots (0.5° × 0.5°) was presented on a white background. During the moving block, the dots moved radially at a speed of 30°/s, reversing direction every second. In each case, a complete cycle of moving and stationary stimuli took 32 s and was presented 8 times (TR = 2 s, TE = 49 ms; 3 mm isotropic voxels, 48 transverse slices). All stimuli were generated using a VSG 2/5 graphics card (Cambridge Research Systems) and participants fixated a central cross for the duration of the scan.

Data were analyzed using FEAT, and GLM analysis was applied to identify voxels that were significantly more active during stimulus blocks than during rest periods. Areas V1, V2 and hMT+ were selected as regions of interest, and Featquery (part of FSL) was used to compute mean percentage BOLD change for these visual areas in each hemisphere.

#### 2.4.4 Statistical analysis of MRI metrics

To determine whether the white matter microstructure differs between the PCA patients and control groups and according to visual deficit, a 2-way ANOVA was performed with main effects of group (patient or control) and hemisphere (ipsi- or contralateral to visual field deficit). A 3-way ANOVA with group, hemisphere and visual area as main effects was performed to identify differences in cortical thickness and percentage BOLD change in functional activation.

## 3 Results

All participants included in the study showed visual field deficits measured with Humphrey perimetry. Fig. 1 gives examples of the field loss for four patients, showing unilateral loss in both the left and right visual fields. P04 and P10, both of whom show advanced PCA, have deficits crossing the midline, affecting both fields. Table 1 gives the field loss for all 10 patients, indicating that 8 of them have greatest loss in the left visual field, although both hemi-fields are affected to a certain degree in all patients. Of the 10 patients only 2 showed evidence of neglect. Both had left hemianopia, one (P07) had left sided neglect the other (P03) right sided neglect. The discordance of side for hemianopia and neglect for P07 suggests that the visual field deficit cannot be explained by neglect.

### 3.1 White matter microstructure is affected in the occipital cortex and corpus callosum

**Fig. 2:**
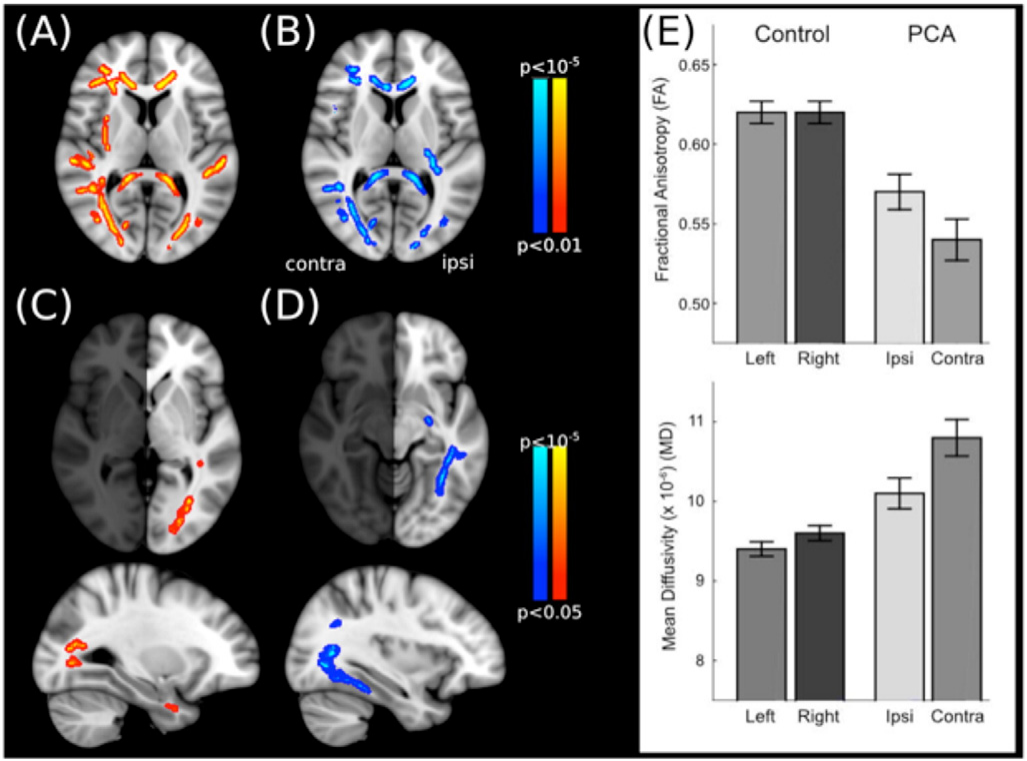
Regions in which the white matter microstructure is significantly different in patients with PCA compared to healthy, age-matched control participants. (A–B) The left image (red) shows the tracts in which FA is significantly reduced, and the right (blue) shows the tracts in which MD is significantly increased. (C–D) Tracts in which the microstructure is significantly more affected in the hemisphere contralateral to the visual field loss compared to ipsilateral. (E) White matter microstructure metrics measured within the optic radiation in each hemisphere separately. Both FA and MD are significantly different from healthy control subjects. Furthermore, the affected hemisphere shows both reduced FA and increased MD in the PCA patients compared to the unaffected hemisphere. (For interpretation of the references to color in this figure legend, the reader is referred to the web version of this article.)

A whole brain analysis was conducted to determine the regions of white matter that showed changes in microstructural properties in the PCA patients compared to healthy controls. Reduction in FA and increase in MD are markers of changes in white matter microstructure. Fig. 2A shows the white matter tracts in which FA was reduced compared to controls. Affected regions are predominantly in the occipital lobe, but also include parts of the corpus callosum; indeed both the genu and the splenium are affected. Increases in MD in PCA patients compared to healthy controls are shown in Fig. 2B. Similar in pattern to the decrease in FA, the major increases are evident in the occipital lobe and both the genu and splenium of the corpus callosum.

To determine the extent to which the abnormality of the white matter microstructure was lateralized, the difference between left and right hemispheres was calculated for each subject's FA and MD images. A group analysis determined the magnitude of this hemispheric difference across the patient group. Fig. 2C shows the regions in which FA was decreased in the affected (right) hemisphere compared to the lesser affected left hemisphere. The optic radiation clearly shows significantly decreased FA in the affected side. Interestingly, Fig. 2D shows the regions in which MD was significantly increased in the affected side compared to the unaffected. In this case the regions are in the occipito-temporal cortex rather than the optic radiations themselves.

Since hemianopia is usually produced following damage to the early stages of the post-chiasmatic visual pathway, the major tract in this pathway, the optic radiation was chosen for detailed analysis. The FA and MD values for the affected and unaffected hemispheres were extracted from patients and compared with the right and left hemispheres of the control group. For both metrics, there was a significant effect of group (FA: F(1,18) = 24.375, *p* < 0.001; MD: F(1,18) = 22.913, *p* < 0.001) indicating a global change in white matter microstructure in the optic radiations. There was also an effect of hemisphere for both metrics (FA: F(1,18) = 14.663, *p* < 0.005, MD: F(1,18) = 22.913, *p* < 0.001), likely driven predominantly by the PCA group. This is evidenced by the significant interaction, particularly for FA (FA: F(1,18) = 11.735, *p* < 0.003; MD: F(1,18) = 9.157, *p* < 0.01). It is evident from Fig. 2E, and planned pairwise comparisons that FA is significantly more lateralized in PCA compared to controls (Controls: t(9) = 0.391, *p* > 0.5; PCA: t(9) = 4.234, *p* < 0.005). The lateralization was present at an individual subject level for the two patients with visual defects in the opposite hemisphere to the other patients – as with the group overall, they both showed greater degeneration in the hemisphere contralateral to the most extensive VF deficit. While PCA patients also show a lateralization of MD (t(9) = − 3.977, *p* < 0.005), a similar effect is evident in the controls (t(9) = − 4.707, *p* < 0.005) likely due to the very small variance.

### 3.2 Gray matter loss is greatest in extrastriate occipital regions

**Fig. 3:**
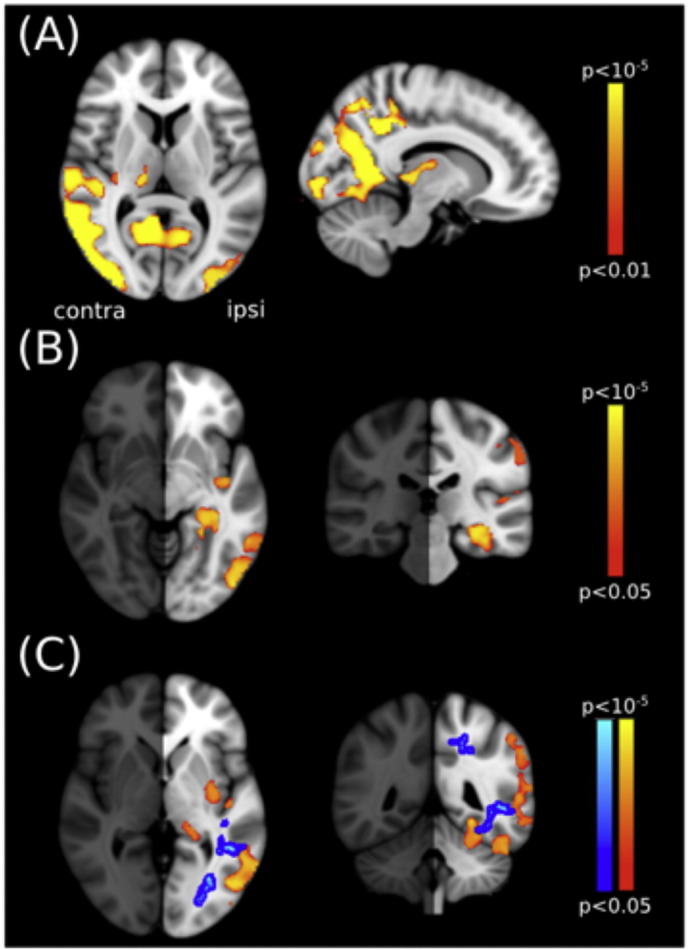
Regions of gray matter that are reduced in PCA patients relative to control participants. (A) The affected areas are predominantly in the posterior portion of the cortex and include lateral and anterior occipital regions. (B) Regions in which the affected hemisphere has greater gray matter loss than the unaffected one. Specifically this lateralization is found in the lateral occipital region and hippocampus. (C) The overlap in lateralized effects of gray and white matter loss. The blue region indicates the increase in MD and red is the gray matter loss. (For interpretation of the references to color in this figure legend, the reader is referred to the web version of this article.)

Voxel-based morphometry was employed to determine differences in gray matter volume between the PCA patients and control subjects. Indeed, Fig. 3A shows the regions in which gray matter is reduced in the PCA patients compared to controls. The most significant regions of reduction are in the lateral and anterior occipital cortex, with loss also evident in the parietal lobe. Furthermore, there is an indication that the gray matter loss is greater in the hemisphere contralateral to the greatest field loss. Indeed the test for asymmetry in gray matter volume between the two hemispheres shows a large difference between the two hemispheres in the lateral occipital regions (− 46, 73, − 10; Fig. 3B).

### 3.3 Hippocampal atrophy is also lateralized

While the medial temporal lobe is less affected in PCA than in classical AD, the laterality analysis indicates significantly greater gray matter reduction in the affected hemisphere (Fig. 3B). Specifically, the loss is centred at MNI coordinates (− 31, − 34, − 15), corresponding to the parahippocampal gyrus region.

### 3.4 Lateralized loss of gray and white matter overlap in the lateral occipital cortex

When the lateralized loss of gray matter and increased MD in the occipital lobe are superimposed, it is clear that they are in a similar region of the occipital lobe (Fig. 3C). In particular gray and white matter appear to have a lateralized pattern of loss in regions linking ventral and lateral regions of the occipital cortex. This may suggest that entire networks of visual processing may be dysfunctional.

### 3.5 Cortical thickness is reduced most in extrastriate visual areas

**Fig. 4:**
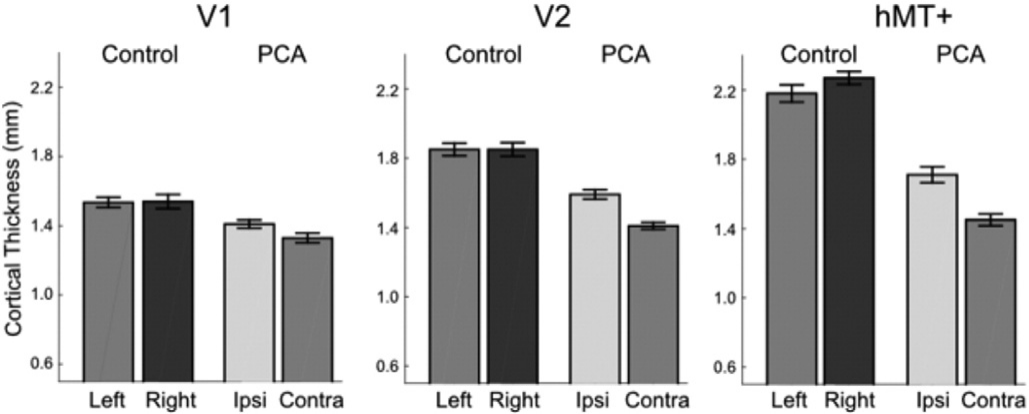
Cortical thickness is reduced in the PCA patients in all visual areas compared to controls. The PCA patients also show hemispheric asymmetry, with the contralateral hemisphere having lower cortical thickness than the ipsilateral hemisphere, the greatest asymmetry being in area V2 and hMT+. Standard errors are shown for all groups.

The mean cortical thickness in visual areas V1, V2 and hMT+ were extracted from Freesurfer and compared between the PCA patients and controls for each hemisphere. Fig. 4 shows the considerable reduction in cortical thickness in V2 and hMT+ in both hemispheres of the PCA patients, although the reduction is significantly greater in the affected hemisphere. These differences were reflected in a highly significant difference in cortical thickness between the two groups (F(1,18) = 98.686, *p* < 0.001) and significant interaction between visual area and patient group (F(2,36) = 79.548; *p* < 0.001). The lateralization of decreased cortical thickness is illustrated by the significant interaction between hemisphere and patient group (F(1,18) = 46.269; *p* < 0.001). Planned pairwise analysis comparing the two hemispheres indicated that in the PCA group both V2 (t(9) = 12.870, *p* < 0.001) and hMT + (t(9) = 5.757, *p* < 0.001) showed a highly significant difference in thickness between the two hemispheres, while V1 was just significant (t(9) = 2.499, *p* = 0.034).

### 3.6 BOLD activity to visual stimulation is reduced across all visual areas

**Fig. 5:**
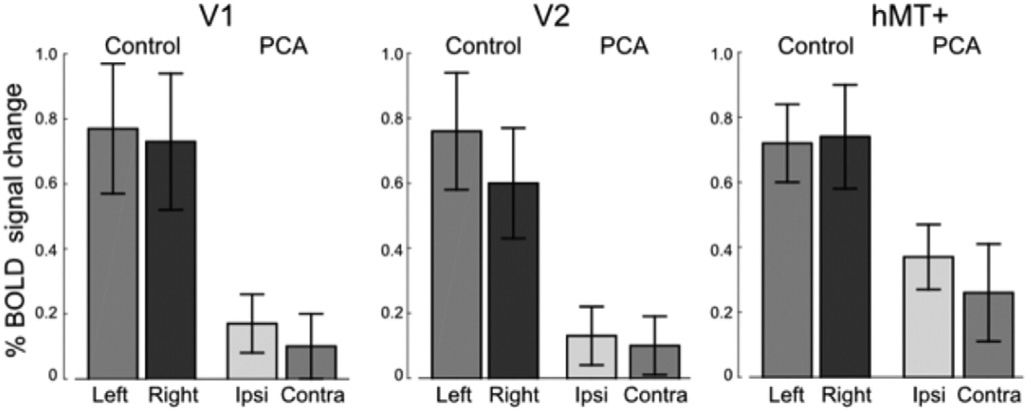
The percentage BOLD signal change to moving, compared to stationary, dots is reduced in the occipital cortex, although the response in both control participants and PCA patients is variable. Activation in the contralateral and ipsilateral hemispheres is similar. Standard errors are shown for all groups.

FMRI data was collected for all PCA patients except P02, who was unable to tolerate this part of the investigation. The activation elicited by moving stimuli was reduced across the occipital cortex in the PCA patient group compared to controls. The percentage BOLD change varies considerably between people, and hence there are relatively large error bars evident in Fig. 5. Nonetheless, the overall response rate between groups differed significantly (F(1,16) = 8.244, *p* < 0.05). There was no significant lateralization of response, with no significant effect of hemisphere (F(1,16) = 3.671, *p* = 0.073) or interactions.

### 3.7 Linking visual fields to neuroimaging measures

**Fig. 6:**
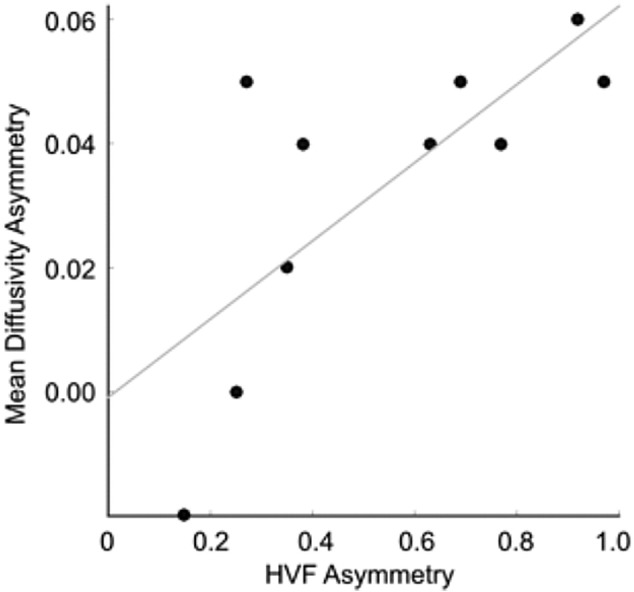
Asymmetry in the visual field deficits of PCA patients is associated with the degree of asymmetry in mean diffusivity in the optic radiation (*R* = 0.752, *p* < 0.01, *n* = 10).

To investigate whether visual field loss could be predicted from measures of neural damage, HVF asymmetry (calculated as (ipsi-contra) / (ipsi + contra)) was correlated with asymmetry in the following measurements: FA and MD in the optic radiation, and cortical thickness and percentage BOLD change in V1, V2 and hMT+. HVF asymmetry was significantly correlated with asymmetry in MD and FA in the optic radiation (MD *R* = 0.752, *p* < 0.01, *n* = 10; FA *R* = 0.624, *p* < 0.05, *n* = 10), and with cortical thickness in area V2 (*R* = − 0.709, *p* < 0.05, *n* = 10). Following correction for multiple comparisons, only optic radiation MD remained significantly associated with the level of asymmetry in visual field loss in PCA patients (see Fig. 6). Note that the values of the asymmetry index for MD in the optic radiation are considerably smaller than those for the HVF. This is because changes in MD are relatively small, only around 10% even when degeneration is significant.

## 4 Discussion

All patients reported in this manuscript showed homonymous hemianopia, in some cases bilateral but in all cases asymmetric. Consistent with previous imaging studies in PCA (Tang-Wai et al., 2004, Whitwell et al., 2007, Singh et al., 2015), neuroimaging indicated that the occipital and parietal lobes are most severely affected.

### 4.1 Hemianopia was in the left visual field in 80% of patients

PCA has previously been reported to affect the right hemisphere more commonly than the left and, in agreement with this, only 2 of the 10 patients considered here had predominantly left hemisphere involvement. Furthermore, where the damage was greater in the left hemisphere the patients showed a right hemianopia as would be predicted.

In a number of studies of PCA, it has been anecdotally noted that while both hemispheres degenerate, atrophy is more pervasive in the right hemisphere (Whitwell et al., 2007, Migliaccio et al., 2012a). As the right hemisphere is noted as being more involved than the left hemisphere in visual-spatial processing (Corballis, 2003), this observation, in combination with the location of the atrophy in the posterior brain regions, is consistent with the higher order visual deficits exhibited by PCA patients. Crutch et al. (2012) have suggested that this represents a selection bias, labelling patients as suffering from PCA if they have visual dysfunction, which is more likely to be a dominant feature of right hemisphere atrophy. On the other hand Kaeser et al. (2015) found 3 out of 10 patients had predominantly left hemisphere atrophy, while damage in the other patients was bilateral. It is therefore not clear whether there is a predilection for the right hemisphere or not in this condition.

### 4.2 Gray matter degeneration and lateralization are maximal in extrastriate regions

Hemianopia due to stroke or trauma results from damage to primary visual cortex (V1 or striate cortex) or the optic radiations, damaging the major pathway from the LGN to the cortex. In contrast, the hemispheric asymmetry present in the occipital cortex of PCA patients is greatest in higher visual areas such as hMT + and lateral occipital cortex.

We have therefore confirmed previous findings that PCA patients show significant degeneration in the occipital lobe, with a tendency towards greater atrophy in the right hemisphere in comparison to healthy age-matched controls. Further analyses investigated whether significant interhemispheric differences exist in the PCA patients, directly comparing the hemispheres ipsi- and contra-lateral to the hemifield with the more severe visual field deficit. Cortical thickness was significantly reduced in extrastriate visual areas (V2 and hMT +) in the contralateral hemisphere, while in primary visual cortex there was minimal cortical thinning, and thickness did not differ between hemispheres. Group analysis also indicated hemispheric asymmetries in white matter degeneration in the visual pathways, with the exception of some regions of the anterior optic radiation. Furthermore, analysis of optic radiation integrity in individual subjects did show a trend for greater mean diffusivity (suggesting loss of fiber integrity) in the contralateral optic radiation in the majority of subjects; this pattern was not present in analysis of FA.

It is notable that in PCA primary visual cortex is relatively preserved and does not demonstrate an asymmetry relating to the laterality of the visual field defect. Indeed specific analysis of area V1 found no evidence of cortical thinning in this region in comparison to controls, in contrast to extrastriate visual regions where cortical loss was extensive. It is more likely, therefore, that the homonymous visual field deficits result from damage to the optic radiations, extrastriate cortex, or a combination of both. This anatomical basis may account for the unusual features of the visual field defects when compared with classical homonymous hemianopic defects resulting from damage to the optic radiations or primary visual cortex.

### 4.3 The optic radiation shows asymmetry in its microstructure

The white matter microstructure shows a widespread reduction in integrity, including the corpus callosum, and occipital regions. However, the lateralization is significant only in the occipital cortex. It is not clear why FA is most lateralized in the optic radiation, while MD is lateralized in the occipito-temporal region. Nonetheless, it is pertinent that the difference in white matter microstructure is only asymmetric in the occipital cortex, not the parietal lobe. Previous work has highlighted different patterns of degeneration in PCA, suggestive of differential effects on occipito-temporal and occipito-parietal regions (Lehmann et al., 2011), but concluded that they are likely part of a continuum. The data presented here suggest a similar conclusion, but additionally that the presence of a visual field deficit may indicate preferential degeneration of the optic radiation and hence basic visual function.

### 4.4 The hippocampus also shows lateralized degeneration

While PCA initially affects visuo-spatial perception, memory dysfunction is present in later stages of the disease. The lateralization of degeneration in the hippocampus according to the most affected visual field suggests that the asymmetry is global rather than limited to the occipital and parietal lobes. Furthermore, this asymmetry is not present in a large meta-analysis of healthy older control adults, patients with mild cognitive impairment and patients with Alzheimer's disease, which actually found that the left hippocampus was smaller (Shi et al., 2009). This asymmetric atrophy of the hippocampus may contribute to the relative preservation of memory function early in the disease.

### 4.5 Functional MRI activity is reduced, but not lateralized

While both white and gray matter show lateralization of degeneration in the occipital cortex, there is little, or no, lateralization of the BOLD activation in the visual areas investigated. The reasons for this are not clear but the variability of the BOLD signal between participants, both healthy and with PCA, may result in subtle differences being difficult to detect.

Relatively few studies of the functional activity in PCA have been undertaken, and until recently, the only studies were case studies (Delazer et al., 2006, Barbarulo et al., 2008, Feldmann et al., 2008), with one demonstrating decreased activation in areas V1 and V2. A recent study investigating visual function in PCA reported that the majority of the patients studied had peripheral field deficits, but none demonstrated hemianopia. Furthermore, unlike the current cohort, the majority of patients showed significant activation to low-level visual stimulation, including mapping of eccentricity (Shames et al., 2015). Rather than low-level visual impairment, their 5 patients exhibited both reduced fMRI activation and behavioral performance on tasks designed to activate the dorsal visual system in addition to reduced fMRI activation to words. Similarly, Gillebert et al. (2015) show that a group of PCA patients is impaired at processing 3D shape information, but without any reported visual field deficits. The finding that loss of gray matter in posterior inferior temporal cortex showed a correlation with performance on 3D structure from shading judgements led the authors to conclude that high-level visual function is affected, rather than lower level disparity processing.

### 4.6 Concluding comments

Neuroimaging studies of PCA have begun to address questions relating to the deficits of both structure and function. However, one of the issues raised by this increasing number of studies is the heterogeneity of degeneration and function. In particular there appear to be some patient groups showing high-level visual deficits, whereas the current patients all show hemianopia contralateral to the hemisphere showing the greatest degeneration. Of particular interest in the current group is the contrast with homonymous hemianopia due to V1 damage, in which extrastriate visual areas are often spared. Thus, while the basic visual deficit is similar, the underlying pathology differs considerable. Further investigation is required to determine whether there are distinct subtypes of PCA with and without hemianopia, and a greater understanding of such deficits will help to improve diagnosis of this enigmatic disease.

## Funding

This work was funded by the Royal Society through a University Research Fellowship to HB. RM held a Medical Research Council Studentship and a Foulkes Fellowship. MMdS was supported by a scholarship from the Brazilian Conselho Nacional de Pesquisa.
